# Complete genome sequence of an ovine ancestral strain of *Mycobacterium avium* subspecies *paratuberculosis* 6756

**DOI:** 10.1128/mra.01207-23

**Published:** 2024-03-05

**Authors:** Franck Biet, Cyril Conde, Thierry Cochard, Fiona A. Mcintoch, Marcel A. Behr, John P. Bannantine

**Affiliations:** 1 INRAE, Université de Tours ISP, Nouzilly, France; 2 McGill University Health Centre, Montreal, Quebec, Canada; 3 USDA-Agricultural Research Service, National Animal Disease Center, Ames, Iowa, USA; Indiana University, Bloomington, Bloomington, Indiana, USA

**Keywords:** *Mycobacterium avium *subspecies *paratuberculosis*, SI type, genome, PacBio

## Abstract

The complete genome sequence of the most ancestral type SI strain of *Mycobacterium avium* subspecies *paratuberculosis* 6756, isolated from a sheep, was determined. The genome was sequenced using PacBio technology, yielding a genome size of 4,830,294 nucleotides with no identified plasmids.

## ANNOUNCEMENT

### Rationale for sequencing

*Mycobacterium avium* subspecies *paratuberculosis* is the causative agent of paratuberculosis, which affects ruminants worldwide (1). Sequencing efforts are still required to better understand the evolution and transmission dynamics of this pathogen (2). The main obstacle to this research lies in the fact that this mycobacterium grows slowly, which explains why very few complete genomes are available and only one for the SI type (3). Thanks to improved long-read sequencing technology, we have been able to obtain the complete assembly of type SI strain 6756.

### Provenance (origin) of the isolate and culture conditions

The *M. avium* subsp. *paratuberculosis* ovine SI-type 6756 strain was isolated from a sheep in New Zealand in 1991 on Herrold’s egg yolk medium with mycobactin at 37°C (4). The strain 6756 was propagated from one colony in Middlebrook 7H9 medium with 0.5% (vol/vol) glycerol, 10% (vol/vol) oleic acid-albumin-dextrose-catalase enrichment medium (Becton, Dickinson, Oxford, Oxfordshire, UK), and 2 µg/mL of mycobactin J (IdVet, France) at 37°C.

### Genomic DNA purification for long-read sequencing, PacBio

D-Cycloserine (Sigma C6880-1G) was added to 15 mL of culture to obtain a final concentration of 50 µg/mL and incubated for 24 h at 37°C. Bacteria were harvested by centrifugation for 15 min at 4,000 *g* and then the bacterial pellet was resuspended with 160 µL of STET buffer [100 mM NaCl, 10 mM Tris-HCl (pH 8.0), 1 mM EDTA, and 5% Triton X-100). One hundred microliters of Lipase (L0777-50mL, Sigma) and 20 µL of lysozyme (100 mg/mL) were added before incubating the sample at 37°C for 2 h under gentle agitation at 900 rpm (Thermomixer C, Eppendorf). Then, 25 µL of proteinase K (MagAttract HMW DNA Kit, Qiagen) was added, and the sample was incubated overnight at 56°C.

Genomic DNA was extracted using the MagAttract HMW DNA Kit (Qiagen) for purifying high molecular weight DNA according to the manufacturer’s instructions.

### Genome sequencing

The genomic DNA obtained was checked by NanoDrop One (ThermoScientific) and concentrated to 59.6 ng/µL by fluorescence measurement using Qubit dsDNA BR Quantification Kit (Broad Range) and sent without size selection to the sequencing company (GENEWIZ, from Azenta Life Sciences, South Plainfield, NJ, USA) using HiFi prep to create library and a PacBio Sequel IIe sequencing platform.

High-fidelity reads with quality values greater than 20 or exhibiting an accuracy of 99% were acquired by the SMRT Link. HiFi reads below 1,000 bp were removed using NanoFilt v2.8.0 (option -l) (https://github.com/wdecoster/nanofilt) (5). Sequencing generated 406,270 reads to reach a sequencing depth of 549×, with an *N*_50_ length of 7,506 bp. The genome was *de novo* assembled by Flye (v2.9-b1768) with the default parameters (6) into a single circular chromosome of 4,830,294 bp, with a GC content of 69.22% rotated to start at dnaA gene using Circlator (v1.5.5) (7) fixstart command and option --genes_fa set with the K-10 dnaA gene. Genome annotation was carried out by NCBI using NCBI’s Prokaryotic Genome Annotation Pipeline (8).

## Data Availability

The sequence was deposited in NCBI’s public sequence database under the accession number CP139433 (BioProject number PRJNA1043460).
